# Express and Implied Terms

**DOI:** 10.1093/ojls/gqac023

**Published:** 2022-10-19

**Authors:** Frederick Wilmot-Smith

**Keywords:** contract law, contractual terms, express terms, implied terms, interpretation, philosophy of private law

## Abstract

Contract terms can be express or implied. But what does that mean? I argue that the distinction can be illuminated by reference to the philosophy of language. Express terms are best understood by reference to the truth-conditional content of the parties’ agreement; implied terms are derived from express terms by a process of reasoning, albeit one aimed at establishing the parties’ commitments.

## 1. Introduction

Contractual terms can be either express or implied. That statement—disarmingly simple, commonplace—is a source of perennial controversy. For example, in *Marks & Spencer v BNP Paribas*, the Supreme Court disavowed Lord Hoffmann’s advice to the Privy Council in *Attorney General of Belize v Belize Telecom Ltd* on the basis of implied terms.^1^ Judges and scholars also continue to debate whether the law should recognise duties of good faith (sometimes under the rubric of ‘relational contracts’)—and, if so, whether the recognition should be via a term implied in law or in fact.^2^

The intractability of these debates should come as no surprise. For one thing, the proper approach is important to practising lawyers (and their clients), who therefore have every incentive to highlight all possible points of disagreement. More important (and certainly more interesting) is the fact that doctrinal disputes rest upon contested but under-explored theoretical premises. The very notion of a contractual term is puzzling, partly because it has been ignored by scholars; even with a sure grasp of the concept of a term, it is not obvious what the express/implied distinction actually consists in.

Any account of implied terms presupposes an account of express terms—though those presuppositions are often silent preludes to any discussion of contractual terms. I begin by presenting my own account of express terms. My overarching claim is that it is best to understand both express and implied terms by reference to the meaning which lends those terms their content. However, the express/implied distinction in law does not supervene on the explicit/implicit distinction in language. Express terms, in other words, are terms whose content derives from both explicit and implicit features of agreements. Thus, when we interpret agreements—ie when we aim to discern what their express terms are—we are sometimes concerned to establish implicit meanings. Implied terms are, I claim, concerned only with a sub-category of implicit meaning.

These distinctions allow us to vindicate the central justificatory claim Lord Hoffmann made in *Attorney General of Belize*, that implied terms seek to spell out the meaning of an instrument (and, therefore, the justification of their enforcement is no different from the justification of the enforcement of express terms). Further, since the meaning of agreements from which implied terms take their content is itself derived from a rule-governed process of reasoning, the otherwise puzzling decision in *Marks & Spencer*—puzzling, because it offers no account of why implied terms arise—is also rendered explicable. The critical practical question is what the rules should be. That will determine, for example, whether the law should recognise duties of good faith—and when.

## 2. Express Terms

### A. Terms and Meaning

When thinking about contracts, we should distinguish linguistic from legal content.^3^ Linguistic content concerns the information conveyed by an action such as (in our context) an utterance or agreement;^4^ legal content is the relevant change in the set of legal propositions resulting from the action under consideration (the oral agreement, the signature on a written document, etc).^5^ The content of the propositions may be the same. For example, a contractual document might state that ‘*A* owes *B* £10’. Its linguistic content might be the same as the legal content it generates: *A* owes *B* £10. But this connection is a contingent fact and should not mask the conceptual point, that the linguistic and the legal are on different metaphysical planes. Perhaps this is too obvious to voice: a lawyer’s job is not to discover the meaning of anything; it is to ascertain the contribution made—say, by an agreement—to the content of law.^6^ But we should attend to the distinction: the language of ‘interpretation’ has led some scholars and judges to think of ‘contractual interpretation’ as, at root, a philological exercise, which can have insidious effects on doctrine.^7^

There is no orthodox account of the nature of an express term, largely, perhaps, because few have ever paused to consider the issue. For example, Peter Benson’s long and thoughtful treatment of implied terms at no point explains what an express term is.^8^ One account seems to be presupposed in the English cases. Lord Neuberger writes that ‘it is only after the process of construing the express words is complete that the issue of an implied term falls to be considered’.^9^ In so saying, he suggested that express terms are concerned with ‘express words’; we can only turn to implied terms when there are no ‘express words’, or when interpretation of those words is exhausted. This formulation is prominent, particularly in the more recent cases.^10^ There are at least three problems with it.^11^

First, since our focus is upon a type of legal content, any analysis which explains terms by reference to ‘words’ is elliptical. Words are not terms: terms have legal content; words do not. Ellipsis is often fine enough, but we should seek more clarity when trying to understand the concept.

Second, the underlying distinctions (‘express’ and ‘implied’) are most readily and commonly used to analyse semantic concepts, like meaning and reference. With a bit of work, it may be possible to apply them to words. Yet once the idea of ‘implied words’ is spelled out, it seems that terms deriving from implied words can be express. Assume, for the sake of argument, that it makes sense to say that words can be implied in cases of ellipsis.^12^ Suppose that a man walks into a shop and holds up a bottle of water: ‘How much [would it cost to purchase this bottle]?’ The shopkeeper replies: ‘Fifty [pence].’ The man pays. There is a contract of sale; its express terms derive from meaning encoded in the words implied (ie those in square brackets).^13^

Third, every term, express or implied, of a written contract must depend in *some* sense on the words used to form or represent the agreement; no conception of implied terms says that a judge can ignore the contractual text.^14^ The crucial question for an account of express and implied terms is the precise relation between the text used and the terms created. Neuberger’s statement does not grapple with that; at most, it reasserts the question. I turn to the question now.

### B. The Nature of Express Terms

#### (i) The core concept

Suppose that two parties, *A* and *B*, agree on the sale of *B*’s boat. They devise a written contract. Clause 1 reads ‘*A* agrees to purchase and *B* agrees to sell all rights, title and interest in the vessel *Frege*’. Ignore the law for a moment. The agreement encodes truth-conditional content, content which determines aspects of its meaning: about, for example, who *A* is, who *B* is and the identity of the vessel. Call all such information ‘semantic’.

Contractual terms are (roughly) those propositions of law made true by the parties’ act of contracting. The core—though, as I explain below, not the entire—concept of an express term is one where the legal content of a particular proposition is provided by the semantic content of the parties’ agreement.^15^ In the above example, the parties’ agreement creates two terms: an obligation on *B* to transfer title of the vessel to *A*; and an obligation on *A* to pay the purchase price to *B*. Those terms are express because the legal content—who the parties are, what they must do—is given by the semantic content of the parties’ agreement.^16^

This account is clearest where the parties agree to a written text, or where the agreement is formed through linguistic means. However, the same account applies to contracts agreed by conduct. As my example of the purchase of the bottle of water illustrates, actions can (alone, or combined with words) be used to communicate an intention, either to make an offer or to accept an offer made.^17^ Where an offer is made by conduct, the offer will give any ultimate agreement its content. We can apply the same distinctions deployed above and below (in my analysis of contracts formed through linguistic means) to such cases. That is because the concepts I use to explain implied terms are not unique to linguistic means of representing content: gestures can have content, and can generate implicatures and presuppositions, too; indeed, the law on implied terms generated through conduct is a fertile source of further study of that very point.^18^

#### (ii) Beyond the core

John Stuart Mill pointed out that

If I say to any one, ‘I saw some of your children today’, he might be justified in inferring that I did not see them all, not because the words mean it, but because, if I had seen them all, it is most likely that I should have said so: though even this cannot be presumed unless it is presupposed that I must have known whether the children I saw were all or not.^19^

To develop and extend the core concept, two features of Mill’s example are worth stressing.

First, there is a distinction between the intuitive truth conditions of what is asserted in an utterance and other propositions conveyed *by* the utterance. We can mark this distinction by talking of what an utterance *asserts* as distinct from what it *conveys*. In Mill’s example, the speaker asserts the proposition that he (Mill) saw at least one of the hearer’s children. If Mill saw no children, we would, intuitively, call his statement false; if he saw one or more, we would think it true. If we assume that Mill sought to ‘make [his] contribution as informative as required’, we can also deduce that he did not see *every* child.^20^ The utterance conveys that information. But Mill does not actually say that. If he did see all the children, perhaps we might charge him with being misleading, but not of lying.

We can denote this distinction, using terminology I have already invoked, by thinking of a divide between ‘semantics’ and ‘pragmatics’.^21^ Although the distinction is famously slippery, it is customarily thought to be something along the following lines: semantics concerns the truth-conditional content encoded in a sentence or utterance (in its relevant context); pragmatics deals with the information communicated by (but not encoded within) the sentence or utterance. Thus, it is natural to suggest that the semantics/pragmatics distinction, whatever it may be, marks the divide in natural language from which lawyers derive the distinction between express and implied terms.^22^

The suggestion should be rejected. Pragmatic inferences can part-constitute express terms; the semantics/pragmatics distinction cannot, therefore, be central to our inquiry. Consider, to illustrate, a case where I say that ‘I will pay you £10 if you deliver the *Frege*’. As a matter of pure semantics, we agree that: if you deliver the vessel, I will pay. This is logically consistent with you *not* delivering the vessel and my paying you the money. Yet the obligations are clearly dependent; the express terms of our contract are: if *and only if* you deliver the vessel, I will pay you. The ‘and only if’, which converts a conditional to a biconditional, is a pragmatic inference.^23^ In this way, the express terms of a contract can depend on pragmatics for their content.

In light of this, an extension to the core concept can be made. The core concept of an express term is one where the content is the parties’ agreement’s semantic content. Like any core, that concept is incomplete. All semantic content-based terms are express; not all express terms’ content is coextensive with semantic content alone (at least as that concept is customarily understood). How, then, can we incorporate this insight into our concept? It is hard to do so without overkill: as explained below, some pragmatic features are better explained as functions of implied terms. It is better to hold onto the core concept, accepting that it has some fuzzy edges. Indeed, if there were a very clear divide between the two concepts, the debate on the nature of implied terms would not have proved so intractable.

The second key feature of Mill’s discussion is as follows. There is an intuitive distinction between explicit and implicit features of an utterance, but this distinction does not map perfectly onto that between semantics and pragmatics.^24^ Implicit features of speech sometimes contribute to the intuitive truth conditions of what is asserted; sometimes not. I will mark that distinction by using the language of ‘implicit content’ for the former features and ‘implicatures’ for the latter.

To see an example of implicit content, suppose that you come to my party and I tell you that ‘all the beer is in the fridge’. If true, this would be quite some party: my fridge would have all the beer *in the universe* ready for my guests. In context, of course, what I said was that all the beer *for the party* is in the fridge. So what I said was true. Yet there are no words to which you could point as expressing the restriction: it comes from the sentence as a whole, in its context of utterance. Here, then, the content in question is both implicit and part of what is asserted by the utterance. There are numerous similar examples in natural language.^25^ A child falls over and cuts herself; the parent reassures the child: ‘You’re not going to die.’ What the parent has explicitly said is false: the child is bound to die. But the proper interpretation of what is said is something like ‘You’re not going to die *from that cut*’.^26^ We can refer to these ideas in the language of ‘implicit content’.^27^ The ‘beer in the fridge’ example is one of restriction of a quantified domain.

An example of an implicature is, as developed below, the implication of Mill’s statement, that he had not seen all the children. The implicature is divined not from any individual word, but from the words used plus more general ideas about speakers’ purposes in communication.^28^

It is characteristic of all implicit features of expression that there is no phrase, word or morpheme denoting the meaning in question. The explicit/implicit distinction is therefore a promising distinction in natural language upon which the express/implied term distinction could supervene. Perhaps this is what David McLauchlan has in mind when he says that express terms are derived from the ‘express meaning [of] the words’.^29^ His notion seems to be this: express terms derive from the explicit meaning of contractual agreements; implied terms derive from the implicit meaning of agreements.

We should reject this suggestion, too. Terms whose content is given by, or partly by, implicit content are usually understood as express. For example, when the *Antaios* was chartered on a three-year time charter, clause 5 of the charterparty stated that ‘on any breach of this charterparty, the owners shall be at liberty to withdraw the vessel’. The arbitrators, High Court, Court of Appeal and House of Lords all agreed that the clause did not mean what it said. Rather than ‘any breach’, they all agreed that the clause meant ‘any *repudiatory* breach’ (depriving the clause of any contributory meaning to the instrument, since the owners had that power at common law).^30^ The case is perhaps best known for Lord Diplock’s incautious statement about ‘business common sense’.^31^ More interesting for our purposes is the fact that no one thought the issue was one of the implication of terms; it was understood to be a question of interpretation.^32^ The example is no different, conceptually, from that of the beer in the fridge. It shows that the distinction between express and implied terms cannot be that between explicit and implicit features of agreements.

The point is of more than theoretical interest. Had the issue been characterised as concerning the implication of terms—an implied limitation, that is, on the scope of the express clause—it is hard to see how the owners’ argument could have failed.^33^ It was not *necessary* to restrict the owners’ right in the way the House of Lords did. This is, then, one way in which apparently rigid rules on the implication of terms can be manipulated: by collapsing implicit aspects of agreements into express terms.

#### (iii) The concept restated

The core concept is helpful enough for most purposes. A more accurate statement is as follows: express terms are those whose content is given by or *closely relates to* the semantic content (including implicit content) of the parties’ agreement. The emphasised text is to include the pragmatic features of agreements—such as that in *The Antaios*—which are customarily thought of as part of express terms.

With this preliminary account in place, we can now turn to implied terms.

## 3. Terms Implied in Fact

### A. The Basic Distinction

The concept of an implied term seems to have emerged by the end of the 17th century, at least within certain commercial cases.^34^ A century later, the concept was entrenched, usually in cases of sale. For example, in *Gardiner v Gray*, Lord Ellenborough said it ‘is an implied term in every such contract’ that goods sold are ‘saleable’ and ‘answering the description in the contract’.^35^ And in *Morley v Attenborough*, Baron Parke considered ‘whether there is an implied warranty of title in the contract of sale of an article’.^36^ By the end of the century, Bowen LJ was able to formulate the various strands into a general principle, that terms are implied ‘to give such business efficacy to the transaction as must have been intended’.^37^

The law has since come to distinguish between terms implied ‘in law’ and those implied ‘in fact’. The canonical statement of this distinction is in *Liverpool City Council v Irwin.*^38^ Lord Cross said that

When it implies a term in a contract the court is sometimes laying down a general rule that in all contracts of a certain type—sale of goods, master and servant, landlord and tenant and so on—some provision is to be implied unless the parties have expressly excluded it.^39^

Such a term is now said to be one implied ‘in law’. ‘Sometimes, however,’ Lord Cross added,

there is no question of laying down any prima facie rule applicable to all cases of a defined type but what the court is being in effect asked to do is to rectify a particular … contract by inserting in it a term which the parties have not expressed.^40^

I will consider terms implied in fact in this section, before turning to terms implied in law in the next.

### B. Implied Terms and Implicature

#### (i) The thesis

In recent decades, both the concept of terms implied in fact and the test for their implication have been the source of a great deal of controversy.^41^ The debate has focused in part on whether the implication of terms involves a ‘different process’ than the interpretation of express terms. For example, Lord Neuberger has said that ‘construing the words used and implying additional words are different processes governed by different rules’.^42^ But this statement is elliptical, for reasons which should now be familiar: we need to move beyond references to words in any analysis. The challenge is to state plainly, without ellipsis or metaphor, what implied terms actually are.

My thesis is that the content of implied terms does not derive from any content asserted by the parties’ agreement; instead, implied terms are derived from express terms through a rule-governed process of reasoning.^43^ To that extent, the Supreme Court was right in *Marks & Spencer* to stress the distinction between interpretation and implication. However, the method of inference from express terms should be linked to the underlying agreement: an implied term should *also* be one to which the parties can be understood to have agreed. For that reason, the board in *Belize Telecom* was correct to downplay the distinction between interpretation and implication. I will develop and explain my thesis in this section by reference to ‘implicatures’, a concept from the philosophy of language; in the next section, I consider the related but distinct concept of presupposition.

Recall Mill’s example, where someone says ‘I saw some of your children today’. Although such a speaker does not say that he did not see all the children, in Paul Grice’s term he *implicates* that fact. In Grice’s canonical example,

A is writing a testimonial about a pupil who is a candidate for a philosophy job, and his letter reads as follows: ‘Dear Sir, Mr. X’s command of English is excellent and his attendance at tutorials has been regular. Yours, etc.’^44^

The content of the letter is that X speaks English well and turns up to class. The natural reading is that the referee wants ‘to impart information that he is reluctant to write down’. The implicature is: ‘Mr. X is no good at philosophy.’^45^ There is, therefore, an intuitive distinction between the proposition *expressed* in the reference letter and the proposition thereby *conveyed*.

The precise mechanism by which implicatures arise is a matter of intense debate amongst philosophers of language and linguists. The orthodox view is probably something like this: we first decode what is said (the proposition expressed, which is concerned, in the above terminology, with what is *asserted*) and then infer from this some further proposition (the proposition conveyed).^46^ The reference letter refers only to the candidate’s command of English and attendance at tutorials, so it is natural to think that one must first decode, roughly, that the candidate has a good command of English and attends tutorials. From that content, combined with context (and certain assumptions about the purpose of the utterance), we can infer that the candidate is hopeless.^47^

Philosophers of language tend to consider unilateral utterances, not the content of agreements. Nevertheless, these distinctions can be applied—albeit with some revisions—to agreements and, therefore, to contracts. Agreements can generate implicatures.^48^ Consider, to illustrate my claim, *Short v Stone*.^49^ The declaration in *assumpsit* stated that

in consideration that the plaintiff, being then unmarried, at the request of the defendant, had then promised the defendant to marry him the defendant, he the defendant then promised the plaintiff to marry her within a reasonable time next after he should be thereunto requested by the plaintiff so to do …^50^

It also stated that, ‘contrary to his said promise’, the defendant married a third party, one Edith Collins. The defendant’s response was that the plaintiff had not alleged a request prior to issue of the suit. The plaintiff demurred to the plea and that was held to be good. The defendant’s breach was not in failing to marry on request, but in marrying the third party; the absence of request was, therefore, immaterial. The further obligation (not to marry anyone else) arises because, although the promisor never said as much, it is natural for a promisee to infer that the promise to marry on request carried with it the further obligation.^51^

Just as the reference writer did not actually say that the candidate was hopeless, it was no part of the explicit content of the promise that the defendant would not marry a third party. Nevertheless, the parties meant the agreement to include the further obligation. This close relation to what is said is important because it demonstrates that implied terms are *not* simply ‘logical implication[s]’ (ie entailments) of express terms.^52^ Suppose that you and I agree that ‘2 + 2 = 5’. A logical implication of our agreement is that we are fools—but we do not agree to *that*.^53^

This account raises two immediate issues: first, how are the implications of an agreement derived? Second: which implications of an agreement do the parties agree to? These issues must be considered together. We need to distinguish between the implications of the parties’ agreement to which it is sensible to say the parties are committed—and those implications which, while logical inferences of the agreement, are ones the parties should not be held to have agreed. To clarify this, we should return to individual utterances. When seeking to ascertain the implicatures of a speaker’s statement, we must assume something about the speaker’s purpose in making the statement. For example, Grice’s ‘cooperation principle’ assumes that speakers aim to make their conversational contribution ‘such as is required, at the stage at which it occurs, by the accepted purpose or direction of the talk exchange in which you are engaged’.^54^ With that principle in hand, it is easy enough to see how the implicature in the reference example is derived.

This shows that implicatures can be derived from express terms only with something like Grice’s cooperation principle: some statement of the parties’ purposes in reaching the agreement they reached. This is not simply a matter of linguistics: background normative assumptions will condition which further propositions the parties are understood to have agreed. Consider, for example, Lord Hoffmann’s statement that

The question of implication arises when the instrument does not expressly provide for what is to happen when some event occurs. The most usual inference in such a case is that nothing is to happen. If the parties had intended something to happen, the instrument would have said so.^55^

This reticence makes sense only against a background where parties are assumed to be (and thought to be justified in being) self-interested. This reluctance would not be shared when considering, by contrast, agreements between friends.^56^ This point is of central importance: a great deal of doctrinal debate regarding implied terms concerns what the background assumptions of contracting *ought* to be. I return to it in more detail below when I consider implied terms of good faith.

#### (ii) Doctrinal evidence

The close relation between implicature and implied terms can be demonstrated by considering three doctrinal features of implied terms. Each feature is explicable once implied terms are understood as legal implicatures.

First, express terms have a logical priority over implied terms. As Lord Neuberger states, ‘until the express terms of a contract have been construed, it is, at least normally, not sensibly possible to decide whether a further term should be implied’.^57^ The reason is that implied terms are inferred from express terms.^58^ This priority aligns with the linguistic sphere, where implicatures are derived from the content asserted.

Some have expressed doubt about this alleged priority of express terms over implied terms. For example, Lord Mance claimed that courts should not

adopt too rigid or sequential an approach to the processes of consideration of the express terms and of consideration of the possibility of an implication … [P]articular provisions of a contract may … give rise to a necessary implication, which, once recognised, will itself throw light on the scope and meaning of other express provisions of the contract[.]^59^

This is doctrinally heretical. Yet it is analogous to the debate concerning the mechanism by which implicatures arise. Stephen Levinson objected that the two-stage Gricean model, where pragmatic rules are combined with what is said to generate further meaning, was circular. ‘What is said,’ he claimed, ‘seems both to determine and to be determined by implicature.’^60^ The idea is that the product of the reasoning process can be used to feed back into the question of what is ‘said’. But if what is ‘said’ is the input to the reasoning process, how can that be so? There is, Levinson suggested, a ‘chicken-and-egg problem’ about priority: neither what is said nor what is implied can be given priority, since each depends upon the other.^61^ Hence his name for this objection: ‘Grice’s circle.’ It does not matter, for our purposes, what the proper resolution of this puzzle is. The point is that the theoretical foundations of the doctrinal dispute are uncovered once we understand implied terms as implicatures.

Second, an implied term cannot contradict the express terms of the contract.^62^ This assumption is explicable on my proposed model of implied terms: the implicature of an agreement cannot be inconsistent with the content of the agreement itself precisely because the implicature seeks to develop the content asserted.^63^

Third, implied terms can be excluded by express provision.^64^ It is, by contrast, unintelligible to talk of ‘excluding express terms’. That contrast—between express and implied terms—is so obvious as to scarcely warrant comment. Yet it is theoretically significant precisely because the signature component of implicatures is that they can be cancelled without amending the content of the utterance.^65^ It makes sense for a referee to write that ‘Mr X’s attendance at tutorials has been regular’ and then to add that ‘he is a superb philosopher’. By contrast, it makes no sense to say: ‘You’re not going to die (from that cut) and you are going to die from that cut.’^66^ Thus the possible express exclusion of implied terms, and incoherence of ‘excluding express terms’, is explicable once implied terms are understood as implicatures.

### C. Presupposition

Conversational implicature is often distinguished from presupposition. The definition of presupposition, and the way in which presuppositions are inferred, are sources of considerable debate in the literature. We can prescind from most of these issues; however, as I describe below, certain legal disagreements are illuminated by knowledge of the wider philosophical debate.

As a general characterisation, we can say that an utterance presupposes some proposition if (and only if) one can reasonably infer from the utterance that the speaker accepts the proposition (and the utterance would not make sense if they did not).^67^ Agreements, likewise, make presuppositions. For example, the contract where ‘*A* agrees to purchase and *B* agrees to sell all rights, title and interest in the vessel *Frege*’ presupposes that there is a (possibly unique) vessel, named the *Frege*, to which *B* holds title. My account to date has explained implied terms as legal implicatures of express terms. Might implied terms also be derived from presuppositions of express terms?^68^ ‘Yes’, I will argue. But the relation between presupposition and implied terms is complex and requires special care.

Let me first justify my ‘yes’. In the Exchequer Chamber’s decision in *Couturier v Hastie*, Parke B states that ‘when there is a sale of a specific chattel, there is an implied undertaking that it exists’.^69^ The content of this implied term derives from an existential presupposition: an undertaking to sell a thing presupposes the existence of the thing.^70^ The structure of this implied term fits my account developed above: the content of the term is derived from the meaning of the express term but is not itself *contained in* the express term’s meaning.

There are, however, three complications to any account of implied terms which makes reference to presupposition. The first is that the presuppositions of express terms can be obligations or conditions and that not everyone understands the conditions as terms of the contract. Parke B’s dictum illustrates a presupposition as an obligation. By contrast, the High Court of Australia observed in *McRae v Commonwealth Disposals Commission* that (in a contract of sale) the existence of the item—ie the same presupposition Parke B held was promised—may be an implied condition precedent to the validity of the obligation to deliver.^71^ It is not easy to explain why presuppositions are construed in such different ways when the express term is the same. To say that it is ‘a question of construction’ for each individual contract whether the presupposition yields a condition or obligation is little more than a conclusory label; it would not explain the reason *why* the analysis is different.

On some views, such conditions (often discussed in terms of ‘common mistake’ and ‘frustration’) are not juristically distinct from implied warranties: they are implied terms like any other.^72^ Yet that view is far from universally accepted; the orthodox account is that common mistake and frustration are legal doctrines, not epiphenomena of the parties’ agreement. This shows that particular care is needed when developing any account of implied terms by reference to presupposition. However, it does not challenge my thesis: consistent with my claims, the content of both the condition and the promise is supplied by the presupposition of the express term; the proper characterisation of the presupposition as a condition is a separate question to an account of implied terms.

The second complication raised by cases of presupposition is that the presupposition of an utterance cannot normally be cancelled felicitously.^73^ Suppose, for example, that *B* promises *A*: ‘I will transfer title in the *Frege* to you.’ This presupposes the existence of the vessel. It would be incoherent for *B* to add ‘but there is no such vessel’. I have already claimed that the legal parallel to cancellability is the parties’ power to exclude implied terms. The fact that presuppositions cannot normally be cancelled is therefore in tension with the legal position that parties are able to exclude implied terms.

But it is only in tension, not strictly inconsistent with the legal position. When an implied term is excluded, the parties cancel not the presupposition, but the warrant as to its truth. That distinction may be subtle, but is important. It is odd (even suspicious), but not a semantic catastrophe, to say ‘I will transfer title in the *Frege* to you but I do not promise that the *Frege* exists’. The oddity, however, serves to highlight a confusing feature generic to cases where implied terms are founded upon presuppositions. Once *B* has promised to transfer title to the *Frege*, if the *Frege* does not exist, *B* will (absent any implied condition on the validity of the obligation) inevitably owe damages for breach of the express terms. An implied obligation to warrant the truth of a presupposition therefore seems unnecessary: a failure of presupposition entails breach of an express term from which the presupposition was derived. This means that it is difficult to square the existence of implied terms founded upon presuppositions with the law’s rule that implied terms must be necessary.

The third complication is that there is an unusually strong urge, when considering terms sourced in presuppositions, to explain the term as express, not implied. This is demonstrated clearly in the High Court of Australia’s discussion of *Couturier v Hastie*.^74^ The same is true of the High Court’s own decision in *McRae*. The Commission accepted the claimant’s tender for the purchase of an oil tanker said to be ‘approximately 100 miles North of Samarai’.^75^ No such vessel existed. The claimant brought a claim for breach of contract and thus had to prove that a contract existed (or, more precisely, that the obligation to transfer title to the vessel was not cancelled by the parties’ common mistake). The High Court of Australia held that the contract was valid and permitted an action for breach. The Court held that the essential question was: ‘What did the promisor really promise?’^76^ In other words, it regarded the question as turning on an analysis of the *express* terms.

This complication may be partly a function of the contested nature of presupposition in the philosophy of language. There is, for example, a debate concerning whether some (and, if so, which) presuppositions are lexically encoded in the primary utterance. That debate suggests one reason why it may be tempting to understand presuppositions as part of express terms: I have argued that express terms are those terms founded upon the semantic content of the agreement; semantic presuppositions can therefore naturally be understood as constituting express terms. Further, and less charitably, the fact that implied terms founded upon presuppositions are difficult to justify as a matter of necessity may also explain why courts consider them as part of the express terms: as I claimed above in the context of *The Antaios*, the distinction between express and implied terms can be manipulated for precisely these reasons.

These complications illustrate that the relationship between presupposition and implied terms is worthy of further inquiry. They are not, however, problems for my account: implied terms are, stated generally, terms derived from the express terms through a process of reasoning which seeks to determine what further terms the parties are (by their commitment to the express terms) committed to.

### D. Payoffs

This analysis helps to illuminate—and sometimes to dissolve—two controversies in the doctrinal and scholarly literature.

#### (i) Interpretation and implication

I began this article by introducing, albeit briefly, two doctrinal debates. The first was the question, raised most clearly in *Attorney General of Belize* and *Marks & Spencer*, of whether implied terms are functions of the parties’ agreements or the court’s rules; the second was the question of whether the law should (or does) recognise implied duties of good faith. Once the proper theoretical basis of implied terms is understood, these debates can be seen as closely interlinked—and various apparent disagreements between the disputants can be dissolved.

We should begin by characterising the first question more carefully. The orthodox account of implied terms is that they are derived from ‘rules’, and that this derivation is a different process from the interpretation of express terms. There are two stages to this analysis. The first is to establish the content of the express terms of the contract. Once that is done, implied terms are derived from the combination of those express terms with doctrinal rules, such as the ‘business efficacy’ rule.^77^ Adherents of this model thus draw a sharp distinction between interpretation of the contract (that is, the elucidation of express terms) and the implication of terms: express terms are logically prior to implied terms; the content of implied terms is derived in a different manner from that of express terms.^78^ The chief rival to this account is Lord Hoffmann’s advice in *Attorney General for Belize*. Lord Hoffmann claimed that ‘the implication of the term is not an addition to the instrument. It only spells out what the instrument means.’^79^ Hoffmann therefore endorsed an agreement-centred account of implied terms.^80^

My analysis shows what is right about both accounts. The parties have not catered for implied terms in the content of their agreement, which is why courts turn to the implication of terms. However, the implication of terms is still tethered to the agreement: they are derived from the express terms through a rule-governed process.^81^ And, importantly, the rules are best understood as attempts to specify the rules of communication in the context of contracting. This shows why the classic ‘rules’ or ‘tests’ for the implication of terms stress the centrality of the parties’ agreement. This is obvious enough in the ‘officious bystander’ test.^82^ But it is also implicit in the ‘business efficacy’ test: the parties are understood to have a master intention, that any contract they entered into will function, and the judges’ aim is to ensure that that intention is fulfilled.^83^ As Lord Wright put this, a term implied in fact ‘must be implied *if the intention of the parties is not to be defeated*’.^84^ These tests’ reference to the parties’ agreement is explained by my thesis: implicatures of an agreement are meaningfully part of what was agreed. The distinction between agreement and rule-governed models is therefore too crude. The supposed distinction between Hoffmann’s and Neuberger’s accounts is more shallow than commonly supposed: both conceptions of implied terms are, to adapt Derek Parfit’s phrase, ‘climbing the same mountain on different sides’.^85^

There are two crucial benefits to this softening of the contrast between agreement and rule-based accounts. First, it helps clarify what is at stake in various doctrinal debates; second, it enables us to improve upon the contemporary doctrinal debate, while understanding why the doctrine is as it is.

To see the first of these benefits, recall that an implicature is derived from a proposition with an additional rule—for example, Grice’s ‘cooperation principle’, introduced above. Such additional rules are functions of the context in which the primary utterance (in the contractual context, the express terms) is made. Crucially, the law’s rules for the implication of terms have a reflexive aspect.^86^ Given the basis of terms implied in fact, those rules must aim to track the parties’ reasonable expectations about the content of their contracts; however, the law’s rules also part-constitute the parties’ expectations, since what the parties are entitled to expect depends in part on what the law allows them to take for granted.

To illustrate this point, consider the contemporary battle over the implication of a duty of good faith.^87^ In his discussion in *Yam Seng*, Leggatt J was concerned to show that the implied term simply represented what the contracting parties would be likely to expect their contract to mean: he argued that the term in question was an implication in fact. Soon after, Leggatt J stated that (on the contract before him in that case) such an implication could be made as an implication either in law or in fact.^88^ I do not seek to analyse the merits of these decisions. My point is simply that contracting parties to long-term contracts will *now* be able to expect something more than the most basic cooperation to which they were previously entitled.^89^ In that way, the court’s decisions affect the underlying maxims from which further implicatures (and, so, implied terms) can be derived.

This example explains why controversy surrounds the proper tests for the implication of terms and why it can be difficult to see what is at stake in the heated debates. There is controversy because implied terms are a key battleground where the question of what contracting parties can legitimately expect, so far as the law is concerned, is answered. These debates can be both at the wholesale level (concerning what the test(s) for the implication of terms ought to be) and the retail level (in an individual case, concerning what those rules demand for a token contract). Since these are, at root, political questions about the proper distribution of power, powerful and impassioned disagreement is predictable. However, it can be difficult to see what is at stake precisely because these political disputes are fought, as it were, through satellite litigation over the proper maxims to govern the derivation of implicatures.

To see the second of these benefits, notice that Lord Hoffmann made two points in *Attorney General for Belize*. He argued, first, that the theoretical basis for the implication of terms was, as suggested in this article,^90^ a matter of interpreting the parties’ agreement.^91^ And he inferred from this, second, that the old tests, such as the ‘officious bystander’, were therefore no more than rules of thumb.^92^ The Supreme Court seemed to reject that analysis. When Lord Neuberger disavowed the advice of the board in *Attorney General for Belize*, he reaffirmed the old test: terms implied in fact arise only where a proposed term is ‘so obvious as to go without saying or to be necessary for business efficacy’.^93^ These, though, are tests for the implication of terms. Lord Neuberger’s judgment offers no conceptual or theoretical basis for those tests—or, indeed, for the implication of any terms. There is, therefore, no rebuttal of the central theoretical premiss from which Hoffmann inferred his conclusion.

This is a puzzle. But we can make some progress, and throw more light on *Marks & Spencer*, if we assume that Lord Neuberger’s quarrel was not with the premiss, but with the inference. Lord Hoffmann disavowed any test for the implication of terms, simply buck-passing the task to the interpretation of language (as he had already buck-passed the task of the interpretation of contracts to the study of language).^94^ However, it is a more complicated task to ascertain implicatures than it is to divine semantic content; one must be attuned to the context of utterance and, especially, the background norms of the parties forming the agreement. The Supreme Court’s essential concern seems to have been a sense that Lord Hoffmann’s approach would make it too easy to imply terms. The concern is, on this analysis, not that the theoretical premiss is false; it is, instead, a fear that the approach would lead busy judges to forget the distinctly legal context of implied terms (inferring implicatures too readily, as one might between friends or in a non-legal context).

There is a more wide-ranging concern here regarding rule design, a concern which is rarely made explicit in judicial discussions. The optimal legal rule will always depend upon both the inherent and the contingent limits of a legal system. The Supreme Court’s judgment in *Marks & Spencer* presupposes that clever counsel might hoodwink the busier or less able judges, leading to unjustified implications of terms. This might be right; it is a partly empirical question. But, even if so, it is entirely consistent with my thesis in this article, which is that Lord Hoffmann’s theoretical premiss was sound.

#### (ii) Implied terms and the fixation thesis

In Lord Hoffmann’s canonical statement, a contract’s content is a function of ‘the meaning which the document would convey to a reasonable person having all the background knowledge which would reasonably have been available to the parties in the situation in which they were *at the time of the contract*’.^95^ This suggests that the contribution a contract makes to the law is fixed at the time of entry *into* the contract. We can call this thesis, that contractual content is fixed at the point of contracting, the ‘fixation thesis’.^96^

This thesis is important for our purposes because there is sometimes an equivocation, apparent in both the judicial and scholarly literature, between talk of implied terms as something nascent within the parties’ contract (and discovered by courts) and something the courts put into the contract.^97^ Consider this passage of Lord Hodge:

Interpretation is not the same as the implication of terms. Interpretation of the words of a document is the precursor of implication. It forms the context in which the law may have to imply terms into a document, where the court concludes from its interpretation of the words used in the document that it must have been intended that the document would have a certain effect, although the words to give it that effect are absent.^98^

The passage begins with a distinction between interpretation from implication: Hodge states that ‘the law’ implies implied terms, in presumed (though silent) contrast to express terms, which Hodge suggests that the parties themselves add. However, the passage concludes that terms will be implied only when the document ‘must have been intended’ to have some particular effect, ie to include the proposition in question, the implied term. He concludes, therefore, that the parties intended that the term be part of the contract.

If implied terms are additions to contractual content, fashioned by a judge, their existence seems to be inconsistent with the fixation thesis. That is obviously true if the implied terms are added by a judge at trial; it is also true if the implied terms are added by legal rules, certainly if those rules ever change after a contract is agreed. Implied terms thus raise the spectre—for those who agonise about these things—of judicial interference in parties’ bargains. These concerns led Lord Hoffmann to stress, in his own judicial treatment of the topic, that the court has ‘no power to improve upon the instrument’.^99^ When there is an implied term, he went on,

it is said that the court implies a term as to what will happen if the event in question occurs. But the implication of the term is not an addition to the instrument. It only spells out what the instrument means.^100^

My proposal explains both the perennial equivocation and why implied terms can be understood to be consistent with the fixation thesis. There is a sense in which the courts add implied terms: the implication of terms into a particular contract depends in part on the wholesale rules governing the implication of those terms, and those rules are within the court’s control. However, part of the court’s aim, in formulating the rules and adding those terms, is to ensure that the legal content of the parties’ contract tracks the set of propositions to which the parties agreed. In that sense, therefore, implied terms are consistent with the fixation thesis.

## 4. Terms Implied in Law

Certain terms arise not as a direct function of the parties’ agreement, but as ‘legal incidents of [certain] … kinds of contractual relationship’.^101^ This kind of ‘term’, now generally known as a term ‘implied in law’, arises as follows. The terms of a token contract are taken to constitute a type of contract (such as a ‘contract of sale’ or ‘contract of employment’).^102^ The type of contract is then conjoined with the law’s general rules on implied terms—given either by the common law or, as in our example, a statute—to produce further content.

Terms implied in fact must aim to replicate a certain inference of the parties’ contracting, and the rules governing their creation must, therefore, be ­agreement-centric. By contrast, the rules governing the implication of terms implied in law need not bear any relation to the parties’ supposed aims or intentions in forming their contract. The modality of this last statement is vital. The rules concerning the implication of terms in law *need not* relate to the parties’ agreements or intentions—but they may. A statute could imply into all contracts for the sale of boats that the vessels be blue. The term would then be implied into all sales, yet its implication clearly does not relate to the parties’ intentions.^103^ A statute could, equally, imply into all contracts for the sale of boats that the vessels be of merchantable quality. Absent the statute, the same term would probably have arisen in fact, as an implicature of contracting parties’ agreements. The important distinction between terms implied in fact and those implied in law thus concerns the plural justificatory reasons the law can deploy when formulating particular rules.^104^

This shows that there is scant commonality between terms implied in law and terms implied in fact.^105^ It is perhaps too late to uncouple these concepts in lawyers’ minds, but it would be better if we could. Indeed, there is a pernicious aspect of their coupling: the use of the same language—‘implied terms’—allows judges some latitude to develop legal policies under the guise of party autonomy. This, certainly, was the way in which the 19^th^-century creations of the law of contract were justified.^106^ The same justificatory slippage is apparent in much of the contemporary debate on duties of good faith: judicial policy has, perhaps, been smuggled into commercial contracts under the guise of terms implied in fact. This may or may not be a good thing as a matter of legal policy; yet, certainly when we consider the doctrine as scholars, it would be a shame if the nuanced distinctions were missed through use of the same label.

Terms implied in law are, instead, far more closely related to legal doctrines such as the power to terminate for breach, the right to contractual damages on breach and the rule that a contract will be cancelled automatically if a frustrating event occurs. Scholars and judges often attempt to repackage these features of the law as contractual terms, often implied terms. The acceptance of those theses waxes and wanes over time: it was once almost universally accepted that frustration arises due to an implied term; now that view is heretical.^107^ These legal doctrines are very similar to terms implied in law: both are ways in which the law adds contractual content beyond that supplied by the parties’ agreement; both can, unless there is some policy precluding it, be excluded by the contracting parties’ agreement. Even so, there are a number of ways in which they differ. First, and perhaps most obviously, terms are implied in law only into specific types of contract: contracts of employment, contracts for the sale of goods and so on. By contrast, doctrines apply to all types of contract, regardless of their characterisation. Second, and perhaps more importantly, terms implied in law must be ascertained before any legal doctrines are applied. One cannot, for example, decide whether a contract for the sale of goods is frustrated on destruction of the subject matter before one has ascertained whether a term implied in law deals with that risk.

So long as this ordering and the distinctions between the concepts is borne in mind, there is no great risk to use of the same language. Even so, common terms for disparate concepts is a familiar problem for lawyers—and we must always, I have argued, be on particular guard against use of a unitary label, ‘implied term’.

## 5. Conclusion

It is only with a good grasp of the concept of express terms that we can begin to think about implied terms. Further, the scope of express terms determines where the more permissive attitude of interpretation runs out and parties are forced to the more rigid rules of implied terms. This means we have good theoretical and practical reasons to think more carefully about express terms. I began this article with such an account and argued that express terms include implicit features of agreements.

I went on to develop an account of what makes a term ‘implied’. I sought to show the sense in both the agreement-based and the rule-governed accounts of implied terms, and the wider considerations (beyond the parties’ agreement) at stake in all such discussions. None of this will end scholarly and doctrinal debates about implied terms. Perhaps, though, it will put them on a surer conceptual footing—and help bring out what, if anything, is at stake in those debates.

## Footnotes

1
*Attorney General of Belize v Belize Telecom Ltd* [2009] UKPC 10, [2009] 1 WLR 1988; *Marks & Spencer plc v BNP Paribas Services Trust Company (Jersey) Limited* [2015] UKSC 72, [2016] AC 742.

2 Compare eg *Yam Seng Pte Ltd v International Trade Corp Ltd* [2013] EWHC 111 (QB), [2013] 1 CLC 662 and *Al Nehayan v Kent* [2018] EWHC 333 (Comm), [2018] 1 CLC 216 with *MSC Mediterranean Shipping Co SA v Cottonex Anstalt* [2016] EWCA Civ 789, [2017] 1 All ER (Comm) 483 [45].

3 The language of ‘legal content’ is most associated with Mark Greenberg: see eg ‘The Standard Picture and Its Discontents’ in Leslie Green and Brian Leiter (eds), *Oxford Studies in Philosophy of Law*, vol 1 (OUP 2011) 69. The phrase ‘legal impact’ is used by Nicos Stavropoulos, ‘Words and Obligations’ in Andrea Dolcetti, Luís Duarte d’Almeida and James Edwards (eds), *Reading the Concept of Law* (Hart Publishing 2013) 139. I understand these terms to signify the same phenomenon, though I have adopted Greenberg’s terminology because I believe it will be most familiar to contract lawyers.

4 This language is also somewhat contentious: see Greenberg (n 3) 47–8.

5 I develop this account in greater detail in Frederick Wilmot-Smith, ‘Term Limits: What Is a Term?’ (2019) 39 OJLS 705.

6 In the United States, scholars sometimes denote the linguistic exercise as ‘interpretation’ and the legal enterprise as ‘construction’: Arthur Linton Corbin, *Corbin on Contracts: A Comprehensive Treatise on the Rules of Contract Law* (West Publishing Co 1951) §534, 7. This language, which seems to originate in Francis Lieber’s *Legal and Political Hermeneutics* (1837), has some antecedents in English contract law, eg *Chatenay v Brazilian Submarine Telegraph Co Ltd* [1891] 1 QB 79 (CA) 85 (Lindley LJ). I have various concerns with the language. Most pertinent to this inquiry is that it allows one to assume, wrongly, that the meaning of texts has a special status in determining their contribution to the law.

7 See Wilmot-Smith (n 5) 717–21.

8 cf Peter Benson, *Justice in Transactions* (Harvard UP 2019) ch 2.

9
*Marks & Spencer* (n 1) [28].

10 eg *Attorney General of Belize* (n 1) [21] (Lord Hoffmann); *Sembcorp Marine Ltd v PPL Holdings Pte Ltd* [2013] 4 SLR 193 (SGCA) [93]; *Trump International Golf Club Scotland Ltd v Scottish Ministers* [2015] UKSC 74, [2016] 1 WLR 85 [33] (Lord Hodge); *Impact Funding Solutions Ltd v Barrington Support Services Ltd* [2016] UKSC 57, [2017] AC 73 [31] (Lord Hodge); *Wells v Devani* [2019] UKSC 4 [28] (Lord Kitchin); *Duval v 11–13 Randolph Crescent Ltd* [2020] UKSC 18, [2020] AC 845 [26] (Lord Kitchin). There are some earlier precursors to this language, eg *MacAndrew v Chapple* (1866) LR 1 CP 643, 648 (Byles J).

11 Lurking here is an obvious pitfall: any account of express or implied terms which explains those concepts using the qualifiers ‘express’ and ‘implied’ is immediately suspect.

12 I am grateful to Matt Mandelkern for discussion on this point.

13 See further *Wells v Devani* (n 10) [59] (Lord Briggs), considering a contract for the sale of a broom, where the ‘essential terms other than price [are] agreed by conduct’.

14 Leonard Hoffmann, ‘Language and Lawyers’ (2008) 134 LQR 553, 571.

15 I prefer to talk in terms of the parties’ *agreement* than their *intention*. This is a complicated issue and I hope to dodge most difficulties, hence my relegation of the point to a footnote. My choice of ‘agreement’ over ‘intention’ would be objectionable only if the content of an agreement can come apart from the content of a group intention. Since no one has engaged seriously with the group intentionality literature and applied it to contract, it is difficult to know whether that is possible.

16 As the example shows, many express terms are only established after the resolution of the expressions whose reference shifts depending upon the context of utterance. See especially David Kaplan, ‘Demonstratives’ in Joseph Almog, John Perry and Howard Wettstein (eds), *Themes from Kaplan* (OUP 1989) 505.

17 See further *The Leonidas D* [1985] 1 WLR 925 (CA) 936 (Robert Goff LJ); *Interfoto Picture Library Ltd v Stiletto Visual Programmes Ltd* [1989] QB 433, 436 (Dillon LJ) and 445 (Bingham LJ).

18 This is an under-explored area in the literature, but see generally Philippe Schlenker, ‘Gestural Semantics’ (2019) 37 Natural Language & Linguistic Theory 735.

19 John Stuart Mill*, An Examination of Sir William Hamilton’s Philosophy, and of the Principal Philosophical Questions Discussed in His Writings* (Longmans, Green & Co 1865) 442.

20 HP Grice, *Studies in the Way of Words* (Harvard UP 1989) ch 2.

21 Charles Morris*, Foundations of a Theory of Signs* (University of Chicago Press 1938) 6.

22 For a hint of this view, see *FSHC Group Holdings Ltd v Glas Trust Corporation Ltd* [2019] EWCA Civ 1361, [2020] Ch 365 [87] (Leggatt LJ, with whom Flaux and Rose LJJ agreed).

23 See generally Michael Geis and Arnold Zwicky, ‘On Invited Inferences’ (1971) 2 Linguistic Inquiry 561; Lawrence Horn, ‘From *if* to *iff*: Conditional Perfection as Pragmatic Strengthening’ (2000) 32 Journal of Pragmatics 289.

24 For a summary of views on the explicit/implicit distinction, see Robyn Carston, *Thoughts and Utterances* (Blackwell 2002) ch 2.

25 See eg the overused and elusive example of Wittgenstein’s ‘game’: Ludwig Wittgenstein, *Philosophical Investigations* (rev edn, Blackwell 2001) 28.

26 Kent Bach, ‘Conversational Impliciture’ (1994) 9 Mind and Language 124, 134.

27 I take this helpful terminology from Paul Elbourne’s *Definite Descriptions* (OUP 2013) 173. Very similar ideas are expressed in different ways: John Perry talks of ‘unarticulated constituents’ (John Perry, *The Problem of the Essential Indexical: And Other Essays* (OUP 1993) 206–25); Sperber and Wilson refer to ‘explicature’ (Dan Sperber and Deirdre Wilson, *Relevance: Communication and Cognition* (Blackwell 1995) 182); and Kent Bach coined the language of ‘impliciture’: Bach (n 26) 126.

28 eg Grice’s maxim of quantity: Grice (n 20) 26–7.

29 David McLauchlan, ‘Construction and Implication: In Defence of *Belize Telecom*’ [2015] LMCLQ 203, 208.

30
*Antaios Compania Naviera SA v Salen Rederierna AB (The Antaios)* [1985] AC 191 (HL).

31 ibid 201.

32 Likewise, see the restrictive interpretation given to the clause in *Bank of Credit and Commerce International SA (In Liquidation) v Ali (No 1)* [2001] UKHL 8, [2001] 1 AC 251 (‘any claims … of whatsoever nature that exist or may exist’).

33 Would any such term have been inconsistent with the express terms? I do not see why it is any more inconsistent that an implied term of (say) merchantability in a contract of sale.

34 John Baker, *Introduction to English Legal History* (5th edn, OUP 2019) 381.

35
*Gardiner v Gray* (1815) 4 Camp 144, 145; 171 ER 46, 47.

36
*Morley v Attenborough* (1849) 3 Ex 500, 509; 154 ER 943, 947.

37
*The Moorcock* (1889) 14 PD 64 (CA) 68.

38
*Liverpool City Council v Irwin* [1977] AC 239 (HL). See generally Sir Kim Lewison, *The Interpretation of Contracts* (7th edn, Sweet & Maxwell 2020) §6.01ff.

39 ibid 258.

40 ibid 259.

41 See *Attorney General of Belize* (n 1); *Marks & Spencer* (n 1).

42
*Marks & Spencer* (n 1) [26].

43 A consequence of this view is that a contract must have at least some express terms; it cannot be composed of implied terms alone.

44 Grice (n 20) 33.

45 ibid 33.

46 eg François Recanati, ‘Embedded Implicatures’ (2003) 17 Philosophical Perspectives 299, 300. Compare Robert Stalnaker, *Context* (OUP 2014) 84–5.

47 The context-sensitivity of these inferences is clear to all who read reference letters from both the United States and the UK.

48 My thesis is even clearer if contracts are best understood, as they were until comparatively recently, as sets of dependent and independent obligations: cf *Kingston v Preston* (1773) 2 Doug 689, 99 ER 437 (KB); *Boone v Eyre* (1779) 2 Wm Bl 1312, 96 ER 767 (Court of Common Pleas). That is because the implicature derives from a unilateral utterance (promise), not a joint statement (ie agreement). For a powerful recent statement of the old view, see Jordan English, ‘The Nature of “Promissory Conditions”’ (2021) 137 LQR 630. Note, too, that the concept of agreement may be reducible to a unilateral speech act (like a promise or an offer): Hanoch Sheinman, ‘Agreement as Joint Promise’ in Hanoch Sheinman (ed), *Promises and Agreements: Philosophical Essays* (OUP 2011); Oliver Black, *Agreements: A Philosophical and Legal Study* (CUP 2012).

49
*Short v Stone* (1845) 8 QB 358; 115 ER 911 (QB).

50
*Short v Stone* (1845) 8 QB 358, 358; 115 ER 911, 911 (QB).

51
*Duval* (n 10) [44].

52 Compare Andrew Robertson, ‘The Foundations of Implied Terms: Logic, Efficacy and Purpose’ in Simone Degeling, James Edelman and James Goudkamp (eds), *Contracts in Commercial Law* (Thomson Reuters 2016) 166.

53 See Stephen Neale, *Descriptions* (MIT Press 1990) 73–4.

54 Grice (n 20) 26. cf Sperber and Wilson’s neo-Gricean account of ‘relevance’: Sperber and Wilson (n 27).

55
*Attorney General of Belize* (n 1) [17].

56 See Adam Kramer, ‘Implication in Fact as an Instance of Contractual Interpretation’ (2004) 63 CLJ 384, 400.

57
*Marks & Spencer* (n 1) [28]. See further *Impact Funding* (n 10) [31] (Lord Hodge); *Trump International* (n 10) [35] (Lord Hodge).

58 Lord Neuberger’s expression is more hesitant, suggesting that a contract can be composed of implied terms alone. That is not possible on my account, since an implied term is conceptually related to express terms.

59
*Trump International* (n 10) [42] (Lord Mance).

60 Stephen C Levinson, *Presumptive Meanings: The Theory of Generalized Conversational Implicature* (MIT Press 2000) 186.

61 ibid 172.

62 This is the fifth of Lord Simon’s rules in *BP Refinery (Westernport) Pty Ltd v Shire of Hastings* (1977) 180 CLR 266 (PC). There are numerous precursors, eg *Lynch v Thorne* [1956] 1 WLR 303 (CA) 305.

63 Compare an ironic statement, where ‘we are asked to understand the opposite of what is said’: Quintilian, *The Orator’s Education, Volume IV: Books 9–10* (Donald A Russell ed and tr, Harvard UP 2002) Book 9.2, 59. Such complications cannot arise in the context of agreements.

64 eg *Geys v Société Générale, London Branch* [2012] UKSC 63, [2013] 1 AC 523 [55] (Baroness Hale): a term might be ‘implied into a class of contractual relationship … unless the parties have expressly excluded it’.

65 See Grice (n 20) ch 3.

66 See Carston (n 24) 124 and 138 (comparing ‘explicature’ and ‘pragmatic inference’ on grounds of cancellability).

67 See generally Robert Stalnaker, *Context and Content: Essays on Intentionality in Speech and Thought* (OUP 1999) ch 2; Scott Soames, *Philosophical Essays, Volume 1: Natural Language: What It Means and How We Use It* (Princeton UP 2008) 93. Particularly given what I say in the text to n 53, presupposition must be clearly distinguished from entailment.

68 For a suggestion along these lines, see Luke Harding and Matthew Harding, ‘Misleading Silence under Australian Consumer Law’ in E Bant and J Paterson (eds) *Misleading Silence* (Hart Publishing 2020) 72–3.

69
*Couturier v Hastie* (1852) 8 Ex 40, 55; 155 ER 1250, 1256 (Exch). It is tempting, but wrong, to see implied warranties of title or authority as analogous: cf s 12 of the Sale of Goods Act 1979; *The Res Cogitans* [2016] UKSC 23, [2016] AC 1034, [39] (Lord Mance). However, these are not presuppositions, strictly so-called; they can, for example, be cancelled quite naturally (and are thus best seen as implicatures, consistent with my primary account of implied terms): see Mackenzie Chalmers, *The Sale of Goods Act, 1893* (W Clowes 1894) 26; s 12(3) of the Sale of Goods Act 1979.

70 Note that this presupposition might not have been common ground until the promise which led to the agreement. See generally Stalnaker’s notion of ‘context set’ in Stalnaker (n 67) ch 5.

71
*McRae v Commonwealth Disposals Commission* (1950) 84 CLR 377 (HCA) 409.

72 JC Smith, ‘Contract, Mistake, Frustration and Implied Terms’ (1994) 110 LQR 400; J English, ‘“Frustration” in Discharge of Contractual Obligations’ (unpublished doctoral thesis, 2022).

73 cf Grice (n 20) ch 17. The exception is where the proposition expressed by the utterance is embedded under an operator, eg a conditional or negation. This point is irrelevant in our context.

74
*McRae* (n 71) 402–5 (Dixon and Fulliger JJ).

75 ibid 396.

76 ibid 407.

77 eg JW Carter and Wayne Courtney, ‘*Belize Telecom*: A Reply to Professor McLauchlan’ [2015] LMCLQ 245, 251 (‘a matter of contract doctrine’).

78 For clear statements, see *Philips Electronique Public SA v British Sky Broadcasting Ltd* [1995] EMLR 472 (CA) 481 (Sir Thomas Bingham MR); *Marks & Spencer* (n 1) [26]. Implied terms could also, on this model, arise from rulings (ie decisions of individual judges or courts on the terms of the token contract). But those rulings will, in the familiar manner, contribute to legal rules on implied terms. See generally John Gardner, *Law as a Leap of Faith* (OUP 2012) ch 8.

79
*Attorney General of Belize* (n 1) [18].

80 See further Kramer (n 56); Stephen A Smith, *Contract Theory* (OUP 2004) ch 8.

81 In Grice’s treatment, ‘what is said’ was combined with a series of maxims; implicatures were the outputs: Grice (n 20) 26–30.

82 See the reply to the ‘officious bystander’ in *Reigate v Union Manufacturing* [1918] 1 KB 592 (CA) 605 (Scrutton LJ) and *Shirlaw v Southern Foundries (1926) Ltd* [1939] 2 KB 206 (CA) 227 (Mackinnon LJ).

83
*The Moorcock* (n 37) 68 (Bowen LJ). By way of analogy, see the ‘master implicature’ of legislation: Timothy Endicott, ‘Interpretation and Indeterminacy: Comments on Andrei Marmor’s Philosophy of Law’ (2014) 10 Jerusalem Rev Leg Stud 46, 55.

84
*Luxor (Eastbourne) Ltd v Cooper* [1941] AC 108 (HL) 137 (emphasis added).

85 Derek Parfit, *On What Matters*, vol 1 (OUP 2011) 419. In his original Tanner lectures, ‘What We Could Rationally Will’, Parfit attributes the phrase to Mill. He probably has in mind a remark attributed to Mill by Lady Amberley: Bertrand and Patricia Russell (eds), *Amberley Papers* (1865) vol I, 373. See too Derek Parfit, *Reasons and Persons* (OUP 1986) 114. I am grateful to Sam Scheffler for help tracing this phrase.

86 My thinking here derives in part from consideration of Andrei Marmor’s orthogonal points, regarding implicature and legislation: Andrei Marmor, *The Language of Law* (OUP 2014) 45ff.

87 The launchpad of the modern debate is now *Yam Seng* (n 2) 120 et seq (Leggatt J).

88
*Al Nehayan v Kent* (n 2) [174].

89 cf *Mackay v Dick* [1881] 6 App Cas 251 (HL).

90 Lord Hoffmann’s precise theory differs from my own. His is clearest in Lord Hoffmann, ‘Anthropomorphic Justice: The Reasonable Man and his Friends’ (1995) 29 The Law Teacher 127, 139; Lord Hoffmann, ‘The Intolerable Wrestle with Words and Meaning’ [1998] SALJ 656, 662.

91
*Attorney General of Belize* (n 1) [18] (implied terms spell out ‘what the instrument means’).

92 ibid [27].

93
*Marks & Spencer* (n 1) [23].

94
*Investors Compensation Scheme v West Bromwich Building Society* [1998] 1 WLR 896 (HL) 912H (Lord Hoffmann).

95 ibid 912H (emphasis added).

96 The language is taken from Lawrence B Solum, ‘The Fixation Thesis: The Role of Historical Fact in Original Meaning’ (2015) 91 Notre Dame L Rev 1, 1 and 15.

97 Once you notice this, it is hard not to see it everywhere: Hoffmann, ‘Anthropomorphic Justice’ (n 90) 139 (‘*the judge* implies a term because it is necessary to make sense of the contract’, emphasis added); Smith (n 80) 280 (saying that terms implied in fact are ‘implied by the court’ *and* ‘implicitly agreed upon by the parties’); Hugh Collins, ‘Implied Terms: The Foundation in Good Faith and Fair Dealing’ (2014) 67 CLP 297, 297 and 317 (referring at one point to ‘judicial intervention’ and at another to whether a term was ‘implicitly accepted’); McLauchlan (n 29) 209 (saying that an implication ‘follows … logically’ from an express term and ‘*is* the meaning’ of those terms); Joanna McCunn, ‘Belize It or Not: Implied Contract Terms in *Marks & Spencer v BNP Paripas*’ (2016) 79 MLR 1090, 1090 (‘the court will “imply a term in fact”, finding that the contract really does provide for the issue, albeit implicitly’).

98
*Trump International* (n 10) [35].

99
*Attorney General of Belize* (n 1) [16]. Compare Patrick S Atiyah, *Essays on Contract* (Clarendon Press 1990) 272: ‘in a large number of cases the court is simply … making law for the parties … and … it is a pure fiction to treat this as a matter of “giving effect to the intention of the parties”’.

100
*Attorney General of Belize* (n 1) [18].

101
*Mears v Safecar Securities Ltd* [1983] QB 54, 78.

102 For the distinction between ‘type’ and ‘token’, see Charles S Peirce, *Collected Papers of Charles Sanders Peirce* (Charles Hartshorne, Paul Weiss and Arthur W Burks eds, Harvard UP 1931) 4.537. For scepticism about the process of characterisation required, see Collins (n 97) 307–9.

103 Notice, though, that there is this commonality: terms implied in law are in one sense derived from the express terms of the contract since those express terms must be construed in order to ascertain whether the agreement is the type of contract to which the general rule, implying the term in law, applies. I am grateful to an anonymous referee for pressing me to clarify this.

104 Some reasons are, of course, better than others: my point here is conceptual.

105 Terms implied by law might over time become, because of the reflexive aspect to agreements set out above, terms implied in fact. If a statute creates a standard for a type of good, parties may come to expect that standard to be a part of their contract. But this point, which is obvious enough, does not undermine the general point about the distinction between terms implied in law and in fact.

106 David Ibbetson, *A Historical Introduction to the Law of Obligations* (OUP 2001) 222. For further discussion, see Wilmot-Smith (n 5) 719.

107
*Davis Contractors Ltd v Fareham Urban DC* [1956] AC 696 (HL) 729 (Lord Radcliffe). Compare, however, Hoffmann, ‘Anthropomorphic Justice’ (n 90) 140 and, more recently, Edwin Peel, *Treitel on the Law of Contract* (15^th^ edn, Sweet & Maxwell 2020) §19-124.

## Notes

I am especially grateful to Alex Georgiou, George Leggatt, Matt Mandelkern, Stephen Neale, Robert Stevens and Adrian Zuckerman for discussion and comments on earlier drafts of this article. I am also grateful to all attendees at presentations at NUS Law and Lionel Smith’s discussion forum.

